# GemSIM: general, error-model based simulator of next-generation sequencing data

**DOI:** 10.1186/1471-2164-13-74

**Published:** 2012-02-15

**Authors:** Kerensa E McElroy, Fabio Luciani, Torsten Thomas

**Affiliations:** 1 Centre for Marine Bio-Innovation and School of Biotechnology and Biomolecular Sciences, UNSW. Sydney, NSW Australia, 2052; 2 Inflammation and Infection Research Group, Evolutionary Dynamics of Infectious Diseases, School of Medical Sciences, University of New South Wales, Sydney, NSW Australia, 2052

## Abstract

**Background:**

GemSIM, or General Error-Model based SIMulator, is a next-generation sequencing simulator capable of generating single or paired-end reads for any sequencing technology compatible with the generic formats SAM and FASTQ (including Illumina and Roche/454). GemSIM creates and uses empirically derived, sequence-context based error models to realistically emulate individual sequencing runs and/or technologies. Empirical fragment length and quality score distributions are also used. Reads may be drawn from one or more genomes or haplotype sets, facilitating simulation of deep sequencing, metagenomic, and resequencing projects.

**Results:**

We demonstrate GemSIM's value by deriving error models from two different Illumina sequencing runs and one Roche/454 run, and comparing and contrasting the resulting error profiles of each run. Overall error rates varied dramatically, both between individual Illumina runs, between the first and second reads in each pair, and between datasets from Illumina and Roche/454 technologies. Indels were markedly more frequent in Roche/454 than Illumina and both technologies suffered from an increase in error rates near the end of each read.

The effects of these different profiles on low-frequency SNP-calling accuracy were investigated by analysing simulated sequencing data for a mixture of bacterial haplotypes. In general, SNP-calling using VarScan was only accurate for SNPs with frequency > 3%, independent of which error model was used to simulate the data. Variation between error profiles interacted strongly with VarScan's 'minumum average quality' parameter, resulting in different optimal settings for different sequencing runs.

**Conclusions:**

Next-generation sequencing has unprecedented potential for assessing genetic diversity, however analysis is complicated as error profiles can vary noticeably even between different runs of the same technology. Simulation with GemSIM can help overcome this problem, by providing insights into the error profiles of individual sequencing runs and allowing researchers to assess the effects of these errors on downstream data analysis.

## Background

Next-generation sequencing (NGS) technologies, such as Illumina's Genome Analyzer [1] and Roche/454's GS FLX [2], produce massive volumes of data. For instance, Illumina's Genome Analyzer IIx can produce up to 640 million 150 bp paired-end reads in a single run [3]. Increasing availability of high volume data is opening new possibilities to researchers. These include assessment of rare variants in viral populations via deep sequencing, metagenomic sequencing of bacterial communities, and pooled resequencing of human chromosomes. Extracting meaningful information from these kinds of sequencing projects is often difficult, however, due to the error rates associated with NGS. Separating true variants from sequencing errors remains challenging. Furthermore, analysis is complicated by an ever-increasing variety of downstream software, and a lack of clear standards [4]. Both selecting the most appropriate sequencing technology, and choosing the appropriate software package and parameter values for data analysis are typically done via a 'hit and miss' approach - a costly exercise, even in the world of 'cheap' NGS.

Simulation of NGS data, followed by software benchmarking, presents an alternative approach. Early published simulators include GenFrag [5]. Its usefulness for modern NGS projects is limited by a simplistic error model, a single input genome, and a lack of quality score information. SamTools [6] also supplies a simulator, however it uses a uniform error rate. A uniformly increasing error rate is used in a slight improvement released as 'dwgsim'[7]. NGS features highly heterogenous error profiles [8,9], so the usefulness of this simulator must be questioned.

There is growing evidence that sequence context (i.e., the nucleotide sequence surrounding a base and the base's position within the read) influences error rates in both Roche/454 and Illumina sequencing [8,9]. This awareness has led to more advanced simulators such as MetaSim and Flowsim [10,11]. While MetaSim generates reads from many input genomes and uses sequence-context error models, it cannot be trained on real data and does not assign quality values to reads, limiting its potential applications. The recent program Flowsim is the most realistic NGS simulator to date, with advanced error modelling and quality scores [11]. However it operates only in 'flowspace' and is therefore entirely limited to simulation of Roche/454 pyrosequencing data. Likewise, the unpublished simulator SimSeq [12] empirically captures some characteristic features of Illumina error models, however only allows a single input genome, does not empirically derive all parameters, and cannot simulate Roche/454 data. ART [13], an unpublished cross-platform simulator, also uses context-dependent error models and does assign quality scores. However it appears limited to a single genome and does not allow training on user's own data sets. Thus there is a need for a realistic, cross-platform NGS simulator, as multiple sequencing platforms are likely to persist, each with their own strengths and weaknesses [14].

Here, we describe GemSIM - a General, Error Model based SIMulator of NGS sequencing data. It uses the generic and standardised formats SAM (aligned reads) [6] and FASTQ (raw reads) [15], thus ensuring GemSIM's applicability to both current and emerging NGS technologies. GemSIM creates empirical error models from real NGS data, facilitating technology-, machine-, and even run-specific simulation. GemSIM considers a sequence-context composed of a window of three bases before the current base, the current base, and one base after the current base (we call this the 'sequence-context word'). GemSIM also assigns realistic, empirically-derived quality scores to simulated single or paired-end reads. It can draw reads from either single or multiple genomes or haplotype sets, making it applicable to deep sequencing, metagenomic, and resequencing projects. We demonstrate GemSIM's usefulness for evaluating error models and benchmarking downstream analysis software by using GemSIM to capture the error profiles of two different paired-end Illumina runs and one Roche/454 Titanium run, and by simulating reads from a set of *in silico *generated *Buchnera aphidicola *haplotypes. We then attempt to identify SNPs using the popular program VarScan [16] and assess the effects of different error profiles and technologies on SNP-calling accuracy.

### Implementation

GemSIM is implemented in Python as a command line package, consisting of the four programs GemErr, GemHaps, GemReads, and GemStats. The GemSIM workflow is as follows:

### GemErr

GemErr generates empirical error models from real data. A SAM format alignment of control data is used as input. A list of polymorphic sites or sites which are known to differ from the reference genome may also be supplied; these sites are then considered to be true SNPs and are ignored during error model calculation.

Reads are sequentially parsed, tracking the total number of reads and read length distributions. For paired-end reads, insert size, whether the read is the first or second read in the pair, and the proportion of properly aligned pairs are also recorded. For each base of each read the following information is then stored: a) nucleotide type and base position in read; b) mismatch or true base for the position; c) indels following the current position; d) preceding three bases in the read; e) following base in the read, and f) quality scores for true and mismatch bases and insert bases. Although it is mainly the sequence preceding the current position that is known to affect error rates [8,9], the following base in the read is tracked to allow accurate simulation of indels within homopolymers. Sequence aligners record these errors either at the start or end of a homopolymer. By taking the following base into consideration, indels are only inserted once within long homopolymers, at the end, rather than potentially multiple times within the homopolymer. Empirical distributions for tracked information are stored to a file and used as error models for input into GemReads.

If a particular sequence-context word is not contained at least some minimum × number of times (default × = 4) within the reference genome, then the model is updated using information based on the longest nucleotide sequence that occurs at least × times in the reference. For instance, if AACTG is missing from the reference, however ACTG can be found five times, then the matrix entry where T is the current position, G is the following position, and AAC are the three bases before the current position is updated by considering all other matrix entries where the two bases before the current position are AC, the current position is T, and G is the following position.

### GemStats (optional)

GemStats takes an error model generated by GemErr, and calculates a set of statistics based on this model - for paired-end reads, models for the first and second read in a pair are considered separately. Statistics reported include the overall mismatch, insertion, and deletion error rates; the error rate for each nucleotide; and the error rate by base position within the read. Additionally, any sequence-context words with an error rate more than two standard deviations greater than the average error rate are also reported.

### GemHaps (optional)

GemHaps takes a genome sequence, and an input command specifying haplotype frequencies and the number of SNPs in each haplotype (here, haplotypes are defined as a group of related nucleotide sequences, each differing by at least one SNP). The positions of mutations are randomly determined, and haplotypes are written to a file for input into GemReads. Alternatively, users may create their own tab-delimited haplotype file (for instance, describing SNPs generated according to an evolutionary model).

### GemReads

GemReads requires as input an error model file (generated by users with GemErr or supplied in GemSIM), a FASTA genome file or directory of FASTA genomes (metagenomics mode), an optional haplotype file (user defined or from GemHaps), and a tab-delimited species-abundance text file (metagenomics mode only). Additionally, the user specifies whether the reference genome(s) are circular or linear, and which quality score offset to use (33 or 64, depending on the required FASTQ output version). The requested number of single or paired-end reads are generated as follows, and output as FASTQ files:

(1) Read length and insert length (paired-end only) are randomly chosen from the empirical distributions defined by the error model (read length may also be set to a static value).

(2) In metagenomics mode, an input reference genome is chosen with probability proportional to the species abundance and genome size.

(3) Read location and direction are chosen at random, and the read is copied from the input genome.

(4) Read is assigned to a haplotype (if supplied) and updated with SNPs, where appropriate.

(5) Errors are introduced according to the error models, accounting for read position, sequence-context word, and 1st or 2nd read in pair (for paired-end reads).

(6) Quality scores are assigned using recorded empirical distributions contained in the error model file.

For paired-end reads, steps 1-6 are repeated, however the insert size is used in conjunction with the previous read position and direction to determine the read location. Our error model also tracks how many read pairs have one read that does not align. We simulate this by generating a read that consists entirely of Ns with low quality scores, forcing it not to map. For instance, for 100 bp paired-end Illumina sequencing data, one read would contain 100 Ns, each with a quality score of 'B' or 2.

## Results and Discussion

### Data processing and performance

We used GemSIM to calculate error models for and simulate reads from three different sequencing runs: Illumina Genome Analyzer IIx with Illumina Sequencing Kit v4 chemistry (Illumina v4); Illumina Genome Analyzer IIx with TrueSeq SBS Kit v5-GA (Illumina v5); and Roche/454 FLX Titanium (Roche/454). For the Illumina simulations, error models were calculated from PhiX control lane data aligned with Novocraft V2.07.06 [17]. Soft-clipping was disabled, reads aligning equally well to two genomic positions were randomly aligned to one of them, and the insert size was set with a standard deviation of 200. All other parameters were given their default values. 94.7% (v4) and 97.5% (v5) of reads aligned. For the Roche/454 simulation, we used aligned plasmid control data from a Hepatitis C Virus study [18]. This alignment was performed using Mosaik V1.1.0021 [19]. The maximum percentage mismatch allowed was increased to 1%, while all other parameters were set as recommended for Roche/454 Titanium. 85.4% of these reads aligned. Simulated reads were drawn from a set of *B. aphidicola *haplotypes, created by GemHaps using the *B. aphidicola *Cc reference genome [GenBank ID: PC000263.1]. The number of simulated reads was five million and one million for Illumina GAII and Roche/454 FLX Titanium respectively. This gave a reference coverage of around 1000×. For consistency, alignment of simulated reads was done using Novocraft for the Illumina data and Mosaik for the Roche/454 data [18]. Memory and runtime data are summarised in Table 1 and show that GemSIM can be run with modest memory requirements (< 1 Gb) on a single CPU/desktop computer within a reasonable time frame. As memory requirements are dictated mainly by the size of the sequence-context word and read length, they are largely independent of the number of reads processed or simulated. Runtime scales linearly with the total number of bases processed or simulated.

**Table 1:** Memory and runtime for Illumina and Roche/454 simulations

Program, data	Memory (Gb)	Runtime (h:min)	Data processed (bases)
GemErr, Illumina v4	0.34	3:15	0.9 × 10^8^

GemErr, Illumina v5	0.34	4:23	1.1 × 10^8^

GemErr, Roche/454	0.39	0:20	1.3 × 10^7^

GemReads, Illumina v4	0.44	3:08	5.0 × 10^8^

GemReads, Illumina v5	0.46	3:10	5.0 × 10^8^

GemReads, Roche/454	0.46	2:45	4.0 × 10^8^

Memory requirements and runtime are given separately for the two main programs within the GemSIM workflow, GemErr and GemReads. Both programs were run on a single Intel Xeon E5530 2.4 GHz CPU. To facilitate comparison, the number of bases processed to create the error models is given for GemErr, while the number of simulated bases is given for GemReads. For the simulations, all reads were generated from the same set of *B. aphidicola *haplotypes (created with GemHaps) using the appropriate error model.

### 5-mer presence and frequency

Our approach to error modelling is dependent on k-mer choice, which needs to be long enough to capture sequence-context information, but also short enough to be represented in the reference genome to be simulated and the control genomes used for error modelling. All possible 5-mers were represented more than four times in the *B. aphidicola *reference genome, while 83 (or 2%) of all 6-mers were found less than four times. Furthermore, more than 90% of all possible 5-mers were found four or more times in both the PhiX and the plasmid genomes, used for modelling Illumina and Roche/454 errors, respectively. Less than 30% of all possible 6-mers were present four or more times in these two genomes, while all possible 4-mers were found more than four times in the plasmid genome, and all but one in the PhiX genome (Table 2). This suggests that a k-mer length of 5 provides an appropriate balance between capturing relevant sequence-context information and the possibility of overfitting the data (with associated wasted run time and memory requirements).

**Table 2:** Number of low-frequency (< 4) k-mers in reference and control genomes

Genome	3-mer	4-mer	5-mer	6-mer
***B. aphidicola***	0	0	0	83

phiX	0	1	98	2918

pGEM-T/HCV plasmid	0	0	28	2979

The fact that most 5-mers are contained within the control genomes used also supports the notion that the derived error models can be used to accurately simulate reads from any unrelated reference genomes. For the 10% of 5-mers not well represented within the control genomes, GemSIM derives an error rate based on the relevant 4-mer (or 3-mer, for the one PhiX 4-mer mentioned above).

### Error analysis

Error models for Illumina v4, Illumina v5, and Roche/454 were analysed with GemStats. Error rates are summarised in Table 3. Striking differences between the error profiles of Illumina v4 and Illumina v5 are apparent, justifying the need for empirical chemistry- or run-specific error models when simulating NGS data. Combining results for the first and second reads in a pair, Illumina v5 had an error rate of 0.31%, five times lower than the error rate of Illumina v4 (1.66%). Illumina v4 also showed large differences between the first and second reads in a pair with second reads having considerably greater error rates. Roche/454 gave the least errors and its mismatch error rate of 0.12% is in line with recently published estimates [8]. Consideration of the top five mismatches within their sequence-context suggests that all runs had some trouble with homopolymers, particularly in G or C homopolymers for the Illumina runs. This problem has been well document for Roche/454 data [8,20], and there is some evidence that homopolymers are also problematic for Illumina data [21]. Our data also supports the known problem with the CGG motif in Illumina sequencing [9,21].

**Table 3:** Error model statistics for Illumina v4, Illumina v5, and Roche/454

	Ill. v4 1^st ^read	Ill. v4 2^nd ^read	Ill. v5 1^st ^read	Ill. v5 2^nd ^read	Roche/454
Overall (%)	0.99	2.40	0.28	0.34	0.12

A (%)	1.23	2.86	0.25	0.33	0.14

T (%)	0.91	2.19	0.34	0.39	0.10

G (%)	0.78	2.00	0.23	0.23	0.12

C (%)	1.12	2.78	0.29	0.41	0.12

1^st ^most freq. (%)	GGG**T**A - > GGG**G**A (4.47)	ACA**A**G - > ACA**C**G (3.94)	GGG**T**C - > GGG**G**C (5.85)	AGG**T**G- > AGG**G**G (3.69)	AAA**C**A - > AAA**A**A (1.07)

2^nd ^most freq. (%)	AGG**T**G - > AGG**G**G (3.71)	AGG**T**G - > AGG**G**G (3.29)	CTC**G**G - > CTC**C**G (5.83)	CGG**T**G - > CGG**G**G (2.7)	CCC**A**C - > CCC**C**C (1.02)

3^rd ^most freq. (%)	CCC**A**A - > CCC**C**A (3.15)	CCC**A**A - > CCC**C**A (3.24)	GGG**C**G - > GGG**G**G (4.06)	GGG**T**G - > GGG**G**G (2.45)	CCC**C**G - > CCC**A**G (0.75)

4^th ^most freq. (%)	CGG**T**G - > CGG**G**G (3.06)	GGG**T**A - > GGG**G**A (3.14)	CGG**T**G - > CGG**G**G (3.65)	GGG**T**C - > GGG**G**G (2.03)	AAA**G**G - > AAA**A**G (0.70)

5^th ^most freq. (%)	GGG**T**G - > GGG**G**G (2.71)	ACA**A**A - > ACA**C**A (2.97)	GGG**T**A - > GGG**G**A (3.20)	CGG**T**C - > CGG**G**C (1.98)	AGG**A**A - > AGG**G**A (0.52)

Insertions (%)	0.000723	0.000935	0.000622	0.001300	0.290000

Deletions (%)	0.000434	0.000482	0.000353	0.000484	0.270000

Values give the error rates for each technology. Several measures of error rate are given, including: overall error rates; average error rate for each nucleotide; error rates for the five sequence-context words most likely to result in mismatches (1^st ^most freq. to 5^th ^most freq); and average insertion and deletion rates. For the top five mismatches, the sequence-context word is given with the actual mismatch base in bold (true sequence - > error sequence).

**Figure 1:**
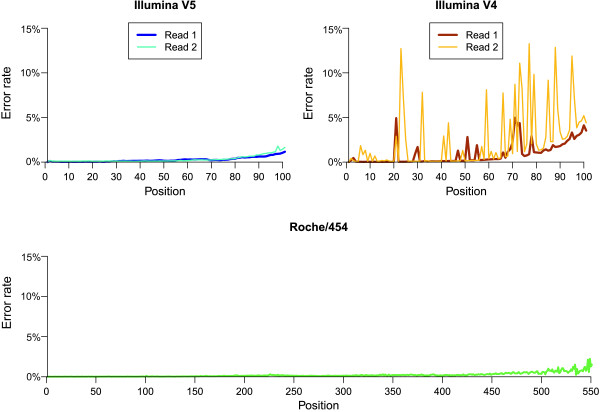
**Error rate by position within read**.

For all three simulations, error rates increased (to varying extents) towards the tail end of the read (Figure 1). While this is a recognised issue for Illumina sequencing, the literature is ambiguous with respect to Roche/454 sequencing data [8,9]. While a positional effect for Roche/454 can be seen in Figure 1, the magnitude of this effect is extremely small when compared to Illumina sequencing data and would probably not be observed if only reads with length < 400 bp are considered. The positional increase in error rate for Illumina v5 is also rather modest, and is only slightly more elevated for the second read in the pair. In contrast, Illumina v4 exhibits a strong influence of base position within the read on error rate, characterised by sudden peaks and a marked increase in error rates near the end of the read.

While insertions were roughly twice as common as deletions in the Illumina runs, in general, indel error rates in both Illumina runs were two to three orders of magnitude lower than in the Roche/454 run. This is consistent with previous findings that mismatches are the predominant forms of error in Illumina, while indels are the most frequent errors in Roche/454 sequencing [20,22], showing that the GemSIM error-modeling approach captures these key characteristics of the different sequencing platforms.

### SNP-calling

For each error model, simulated reads were drawn from a set of five haplotypes derived from the *B. aphidicola *reference genome, with frequencies of 1%, 3%, 5%, 7% and 84%. The four low-frequency haplotypes each contained 100 randomly placed SNPs and the same haplotypes (with their associated SNPs) were used for all simulations and downstream analysis.

SNP-calling was performed using VarScan v2.2.3 [16] on pileup files generated with SamTools v0.1.12a [6]. As we were interested in identifying low-frequency true SNPs and associated false positives (errors), the minimum variant frequency parameter was set to zero. Minimum coverage was set to 100 and the minimum number of reads supporting each SNP was set to five. As VarScan largely depends on individual base quality scores to distinguish between true SNPs and sequencing errors, we varied the minimum average quality (M.A.Q.) parameter from 10 to 40 and investigated its interaction with specific error profiles and SNP-calling accuracy.

SNP-calling was highly accurate for SNPs with a frequency > 3% for all sequencing platforms simulated. Roche/454 showed a slightly lower true positive rate (i.e. an increased false negative rate) than the Illumina simulations. When using VarScan with a M.A.Q. of 20, 379 out of 400 SNPs were identified (86, 97, 97, and 97 for frequencies 1, 3, 4 and 7%, respectively). In contrast, all 400 SNPs were identified from both Illumina simulations. Upon closer inspection, 11 of the false negatives with true frequency of 1% failed to be supported by five reads. All the remaining false negatives were associated with homopolymer indel errors. Inspection of the pileup file showed these SNPs were contained in the data; however VarScan reported them as indels instead.

Any inaccurate SNP calls within +/- 1% of a known haplotype frequency were classed as false positives. For Illumina v5 and Roche/454, all false positives had a frequency under 1% (+/- 1%). Illumina v4 showed a drastically increased false positive rate, as can be expected from the higher average error rate of this run. Despite this, false positives were still restricted to under 3% (+/- 1%) population frequency. As M.A.Q. was increased, however, some false positives with a true frequency of 1% (+/-1%) were now given a frequency by VarScan of 3% (+/-1%). This can be seen as spikes in the frequency = 3% graph, between M.A.Q. 30 and M.A.Q. 40. This can be understood by considering how the M.A.Q. parameter works. The VarScan manual states that M.A.Q. is the 'minimum base quality at a position to count a read' [23]. This means VarScan uses M.A.Q. to select a subset of reads from which to make a call. Thus increasing M.A.Q. reduces the sampling of reads, which in turn reduces the accuracy of the SNP frequency calculation when frequencies are small. This highlights the need for a detailed understanding of any chosen data and analysis pipeline and the value of performing benchmarking with simulated data.

**Figure 2:**
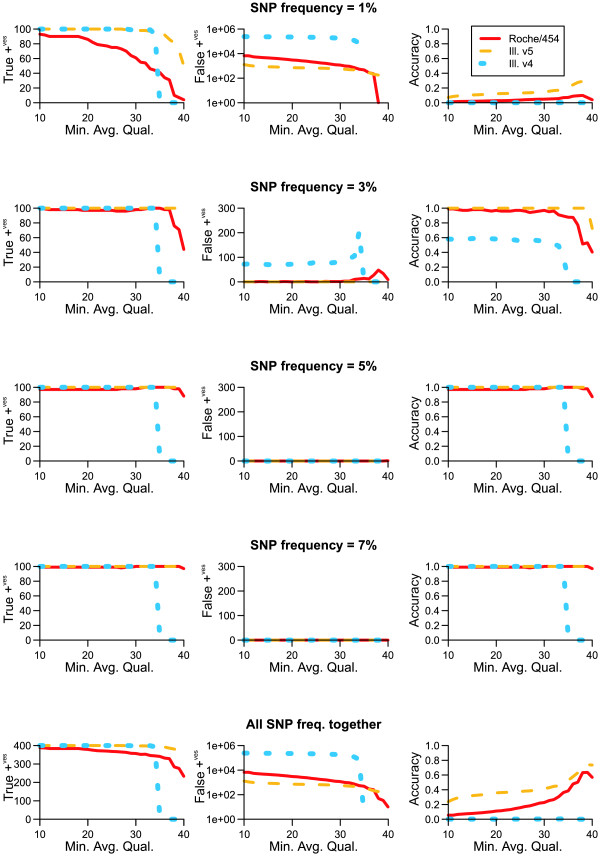
**True positives, false positives, and accuracy for increasing values of M.A.Q**. Graphs for true positives and false positives are in absolute numbers, while accuracy is on a scale from zero to one. (Accuracy is defined as (true positives)/((total SNP no.) + (false positives)). One equals perfect accuracy.) False positive graphs for 'SNP frequency = 1%' and 'all SNP freq. together' are on a logarithmic scale. For false positive graphs, any false positives within +/-1% of the specified frequency are included in the graph.

VarScan also has a minimum coverage parameter and its strong interaction with the M.A.Q. parameter is shown by Table 4. When calculating coverage, VarScan only 'counts' bases that have a quality above a given M.A.Q and thus coverage effectively decreases as M.A.Q. increases. This interaction explains why a M.A.Q. value of 39 gives the most accurate overall results for Illumina v5, however results in no SNPs being called for Illumina v4. As we set the minimum coverage to 100, any genomic position where VarScan counts less than 100 reads will be ignored. Using a M.A.Q. of 39, for the Illumina v5 simulation less than 0.003% of the genome is ignored, whereas for Illumina v4 100% of the genome is ignored. Although increasing M.A.Q. decreases false positives, there is a clear trade-off between decreasing false positives by eliminating low-quality bases and increasing false negatives by disregarding good data. This again reinforces the need to understand individual sequencing runs, even if they originate from the same technology (in our case, the sequencing runs used to create error models for the two Illumina simulations were performed by the same technician on the same machine). Without simulation, it would be difficult to choose an optimal M.A.Q. value and almost impossible to interpret any findings. Following this simulation, for a *B. aphidicola *sequencing experiment resembling our Illumina v5 simulation a M.A.Q. of 39 will give confident and accurate results. Furthermore, we expect to identify 100% of SNPs with frequency > = 3% and 71% of SNPs with frequency of 1% (see Figure 2). We also expect that across the length of the genome, 101 false positives with frequency of 1% (+/-1%) will also occur.

**Table 4:** Number of genomic sites considered by VarScan for increasing values of M.A.Q

**M.A.Q**.	Illumina v4	Illumina v5	Roche/454
10	416376	416380	416374

11	416376	416380	416374

12	416376	416380	416372

13	416376	416380	416370

14	416376	416380	416369

15	416376	416380	416368

16	416376	416380	416365

17	416376	416380	416361

18	416376	416380	416357

19	416376	416378	416357

20	416376	416378	416353

21	416376	416378	416353

22	416376	416378	416349

23	416375	416378	416345

24	416375	416378	416340

25	416375	416378	416336

26	416375	416378	416328

27	416375	416378	416323

28	416375	416377	416317

29	416375	416377	416312

30	416375	416377	416303

31	416374	416377	416292

32	416372	416376	416284

33	416371	416376	416271

34	416368	416376	416260

35	14370	416376	416251

36	0	416375	416233

37	0	416375	416226

38	0	416374	416110

39	0	416368	416076

40	0	416358	415943

M.A.Q gives the minimum quality a base within a read must have for VarScan to 'count' it. At each position with the genome, there must be at least 100 bases (from 100 reads) with a quality above the value of M.A.Q, for the genomic position to be considered by VarScan when scanning for SNPs. For example, a value of zero means there were no sites within the reference genome where there were more than 100 aligned bases with a quality score greater than the specified M.A.Q.

## Conclusions

By considering errors within their sequence-context in real data, GemSIM captured known features of Illumina and Roche/454 error profiles, thus validating our approach. GemSIM therefore facilitates independent, objective, and comparable simulation of both Roche/454 and Illumina sequencing data. Analysis of the error models created by GemSIM also provided new insights into error profiles, specifically that there were substantial differences between the error profiles of the Illumina v4 and Illumina v5 sequencing runs. While it is not clear whether this difference is due to the change in chemistry or to some other factors, it does show that sequencing runs can vary substantially, even when performed by the same sequencing provider using the same machine. Furthermore, differences between these error profiles have a substantial impact on downstream analysis, as shown by our study of SNP-calling accuracy in simulated data.

Our findings call for empirically derived, run-specific error models in sequencing simulation. GemSIM meets this need with a set of python scripts that can be run on a standard desktop computer. By allowing analysis of run-specific error models created from the user's own data, GemSIM helps researchers to identify unique features of their data - understanding of which may be invaluable for downstream data analysis. Error-model analysis also facilitates quality control and identification of 'bad quality' sequencing runs, such as the Illumina v4 run described here. Finally, simulation of sequencing data based on empirically-derived error models allows researchers to choose the most appropriate sequencing platform for their project, assess the impacts of errors on downstream data analysis, and objectively interpret any findings.

GemSIM is most suited to simulating resequencing or metagenomic projects where known reference sequences exist, as it relies on the presence of a reference sequence to initially generate reads (error models are then superimposed on top of the read). For example, GemSIM can be used in a deep resequencing project to establish at which coverage any further sequencing may not provide any increase in SNP detection accuracy. GemSIM may also prove itself valuable in developing and benchmarking de-novo assemblers, by assessing how well a known genome can be reconstructed from simulated reads. By providing a manually modified reference genome to GemSIM, users could also simulate reads to assess the detection of large genomic rearrangements via de-novo assembly.

Future improvements to GemSIM may include increasing the number of bases tracked before the current position during error model construction, as it is possible that error profiles are even more heterogenous than reported here. For Roche/454 sequencing, indel errors are known to increase with increasing homopolymer length, while there is evidence to show that Illumina sequencing accuracy can be influenced by the sequence up to 10 bases before the current position [9]. Currently, the number of bases before the current position is limited by both memory requirements and the need for the sequence-context word to be present in the control dataset. With future improvements in computing power and memory handling, it will be feasible to allow users to optionally increase the sequence-context word length, when appropriate.

As new sequencing technologies emerge, we will also continue testing and developing GemSIM for compatibility. Recently released platforms, such as the Illumina's MiSeq and Pacific Biosciences RS system, can be readily assessed as they are compatible with the two generic formats required by GemSIM, FASTQ and SAM.

The error models described in this paper are provided with the GemSIM package as generic technology-specific error models, for users who do not have access to control data. New error models for different platforms and chemistries will be supplied, as they become available.

## Availability and requirements

**Project name: **GemSIM.

**Project home page: **http://sourceforge.net/projects/gemsim/

**Operating system(s): **platform independent.

**Programming language: **Python 2.6

**Other requirements: **Numpy, Python 2.6

**License: **GNU GPL v3.

**Any restrictions to use by non-academics: **none.

## Competing interests

The authors declare that they have no competing interests.

## Authors' contributions

KM wrote the GemSIM code, KM, FL and TT participated in data analysis, KM drafted the manuscript and FL and TT revised it, and KM, FL and TT contributed to study design and conception. No funding bodies contributed to study design or data analysis. All authors read and approved the final version of the manuscript.
